# 2017年晚期非小细胞肺癌患者登记研究第一次研究者会议报道

**DOI:** 10.3779/j.issn.1009-3419.2017.11.13

**Published:** 2017-11-20

**Authors:** 丽 马, 树才 张

**Affiliations:** 101149 北京，首都医科大学附属北京胸科医院，北京市结核病胸部肿瘤研究所肿瘤内科 Beijing Tuberculosis and Thoracic Tumor Research Institute, Beijing Chest Hospital, Capital Medical University, Beijing 101149, China

**Keywords:** 肺肿瘤, 诊断, 治疗, 预后, Lung neoplasms, Diagnosis, Therapy, Prognosis

2017年10月29日，由北京协和医院呼吸内科王孟昭教授发起的晚期非小细胞肺癌患者登记研究第一次研究者会在中国首都北京成功召开，北京肿瘤防治研究会（Cancer Prevention and Treatment Research, CAPTRA-LUNG）会长、首都医科大学附属北京胸科医院胸外科李福根教授也对该研究的开展予以肯定。研究的参与单位包括北京协和医院、北京肿瘤医院、首都医科大学附属北京胸科医院、北京大学第一医院、北京大学人民医院、北京医院、首都医科大学附属北京朝阳医院、北京大学第三医院、中国医学科学院肿瘤医院、首都医科大学附属北京世纪坛医院、四川大学华西医院、河北医科大学第四医院、山西省肿瘤医院、内蒙古包头市肿瘤医院、中国医科大学第一附属医院、哈尔滨医科大学附属第二医院，共16家医院，包括30余人参加此次会议，讨论研究方案和研究设计。研究的目的在于通过此项登记研究，以肺癌精准防控诊疗需求为导向，通过整合优质资源，建立具有统一标准的晚期非小细胞肺癌专病队列，建成高效肺癌发病追踪系统，形成可共享临床诊疗信息库，形成患者合理的长期管理机制，为实现晚期非小细胞肺癌精准诊疗大数据平台做支撑。由于我国针对肺癌精准医学研究面临的整体设计不足、重复分散建设、资源共享有限等问题，本项目的开展正是通过资源整合，建立具有统一性、标准性和代表性晚期肺癌专病队列，实现资源共享，为精准化诊疗、药物选择、疗效预后评价、生物标志物的发现、检测和推广等方面的研究和临床应用积累有价值的完整的原始基础数据，为后续研究起到引领和推波助澜的作用。

此研究的团队均为诊疗实力强、患者群稳定的医院，此前有共同承担项目的经历，能够保证诊疗规范性、数据的准确性、均一性、统一性。此外，研究项目还纳入经验丰富的数据库管理团队思派北京网络科技有限公司以及《中国肺癌杂志》和《*British Medical Journal*》杂志社团队协助进行大数据分析和医学统计学指导，保证数据的可利用性和研究的流行病学和统计学价值，利于大数据为今后的基础和临床科研工作做保障。

此外，会议针对2017年10月15日-18日由国际肺癌研究协会（International Association for the Study of Lung Cancer, IASLC）承办的第18届世界肺癌大会（World Conference on Lung Cancer, WCLC）关于国际上胸部肿瘤的最新研究进行了广泛的学术交流和深入探讨。以“BEST of WCLC”肺癌专家论坛形式进行探讨，会议上通过中国医药教育协会（Chinese Education Medical Association, CEMA）肺部肿瘤专委会及全国肺癌规范化治疗优秀示范中心（Center Of Excellence, COE）项目委员会认证，授予北京大学人民医院胸外科、北京大学第一医院呼吸内科和北京医院肿瘤内科“全国肺癌规范化治疗优秀示范中心”的牌匾。旨在以区域性中心和省市级分中心为单位向下级医院辐射，通过搭建学术交流平台、开展医生规范化培训、建立多学科诊疗团队等多种方式不断规范和提高肺癌诊治水平，并对患者进行科普教育和心理支持，逐步提高患者生存率及生活质量。中国医药教育协会肺部肿瘤专委会主任委员、上海胸科医院肺内科主任姜丽岩教授致辞，详细介绍了COE项目的宗旨、意义以及目前的成果。以上海市胸科医院为全国示范中心，下属全国5大区域示范中心，包括25家分中心，覆盖全国210家医院。以区域性COE中心和省市级分中心为单位向下级医院辐射，建立上下联动的肺癌诊治绿色通道，充分发挥核心医院和专家的作用，提高中国肺癌治疗水平。成为会议的亮点之一。

会议的另一个亮点是，来自不同医院的专家以及青年学者们共同盘点和讨论了2017年肺癌诊疗的新进展，包括抗血管生成治疗的进展、基因突变的治疗进展、免疫治疗和液体活检的进展等，另外还包括真实世界大数据的相关研究实力，为研究者利用大数据开展研究项目提供新的思路。

本项目拟在2017年12月完成项目方案的修订工作，并开始入组患者，期待研究项目的顺利开展，欢迎各位专家提出宝贵意见。

